# Supplementary material for: EFHD2 suppresses intestinal inflammation by blocking intestinal epithelial cell TNFR1 internalization and cell death
Source: Nat Commun. 2024 Feb 12;15:1282. doi: 10.1038/s41467-024-45539-x (PMC10861516; doi:10.1038/s41467-024-45539-x)
Supplement: Supplementary file 1 — Supplementary information [file 41467_2024_45539_MOESM1_ESM.pdf]

Supplementary information

Supplementary figures 1-8;
Supplementary tables 1-5.

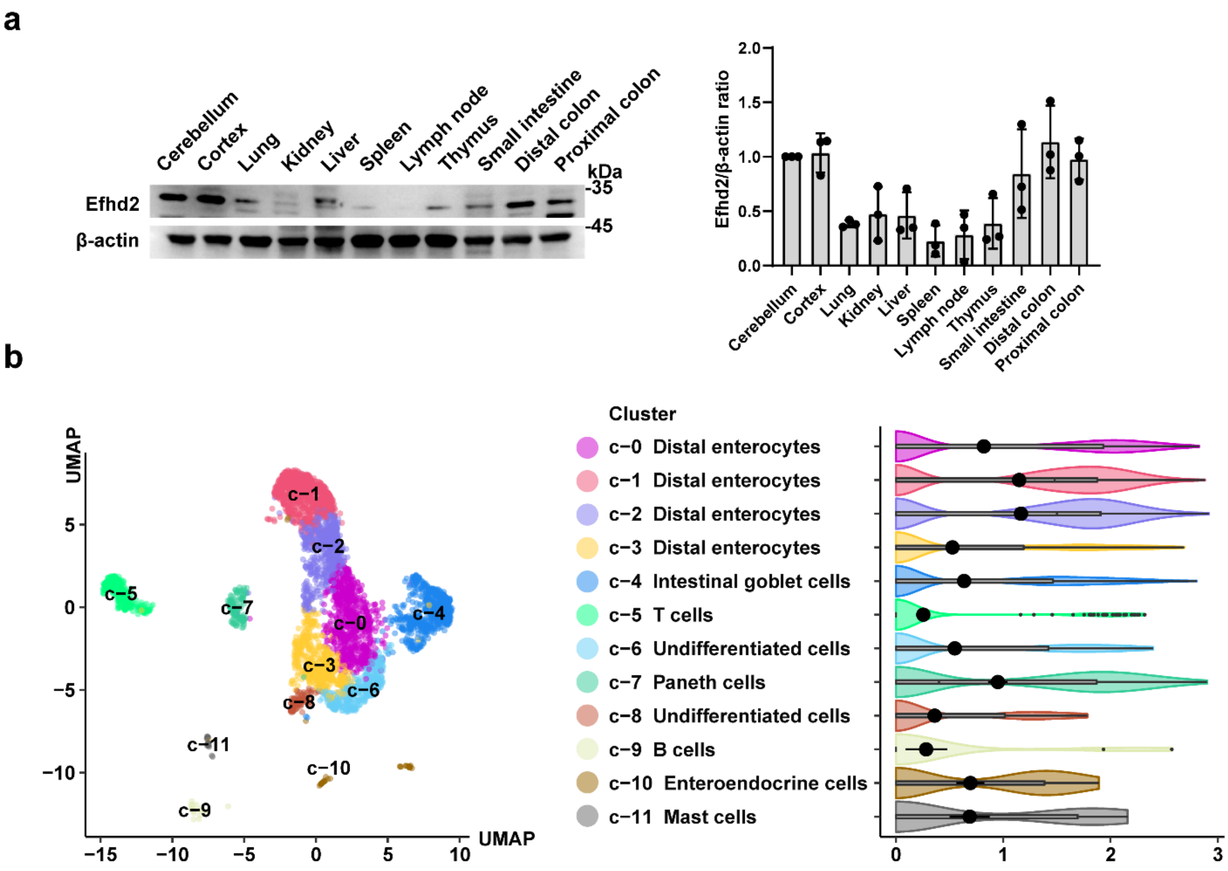

Supplementary figure 1. Efhd2 is highly expressed in intestine tissue and epithelial cells. a Western blot analysis and quantification of tissue lysates of cerebellum, cortex, lung, kidney, liver, spleen, lymph node, thymus, small intestine, distal and proximal colon from C57BL/6J mice. n = 3. Data are representative of three independent experiments; error bars show means ± s.d. **b** Re-analysis of the publicly available single-cell RNA sequencing dataset (GSE116222; https://www.ncbi.nlm.nih.gov/geo/query/acc.cgi?acc=GSE116222) for *EFHD2*

expression levels in multiple cell types from normal human colon tissue. Source data are provided as a Source Data file.

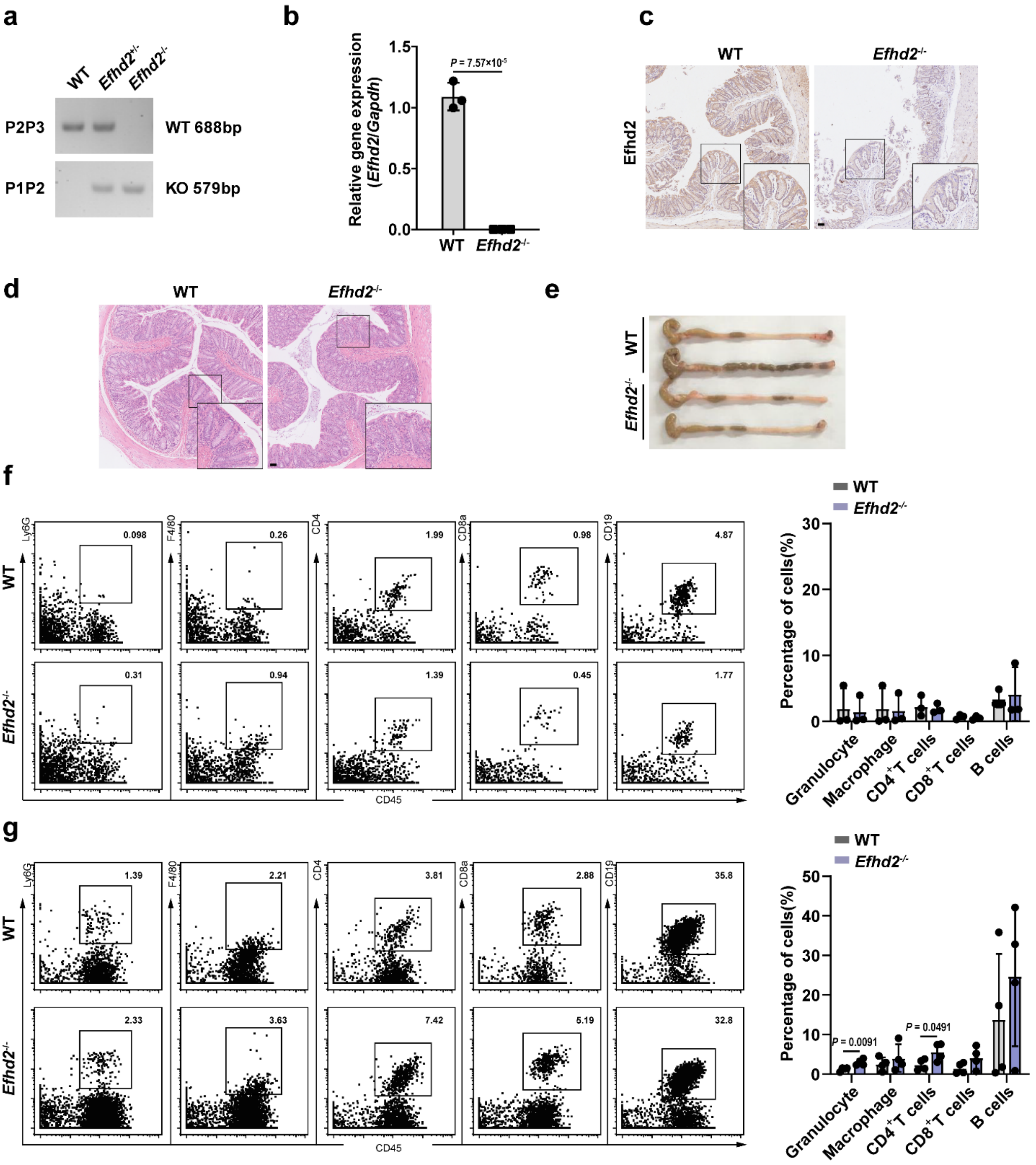

Supplementary figure 2. *Efhd2* deficiency exacerbates colonic inflammation after DSS administration but not at the steady-state condition. a-c Characterization of *Efhd2*-deficient mice by genotyping (**a**), qPCR (**b**) and immunostaining for Efhd2 (**c**) of mouse distal colon tissues from WT and *Efhd2*$^{-/-}$ mice. n = 3 in **b**. **d, e** Representative

images of H&E staining (**d**) and colon morphology and lengths (**e**) of distal colon of WT and *Efhd2*$^{-/-}$ mice at the steady-state condition. Scale bars: 100 μm. **f, g** CyTOF analysis of the proportion of isolated intestinal immune cells from the lamina propria of WT and *Efhd2*$^{-/-}$ mice before (**f**) (n = 3) and after (**g**) (n = 4) DSS administration on day 7. Data are representative of three independent experiments; error bars show means ± s.d. *P* values were determined by unpaired two-tailed *t*-test. Source data are provided as a Source Data file.

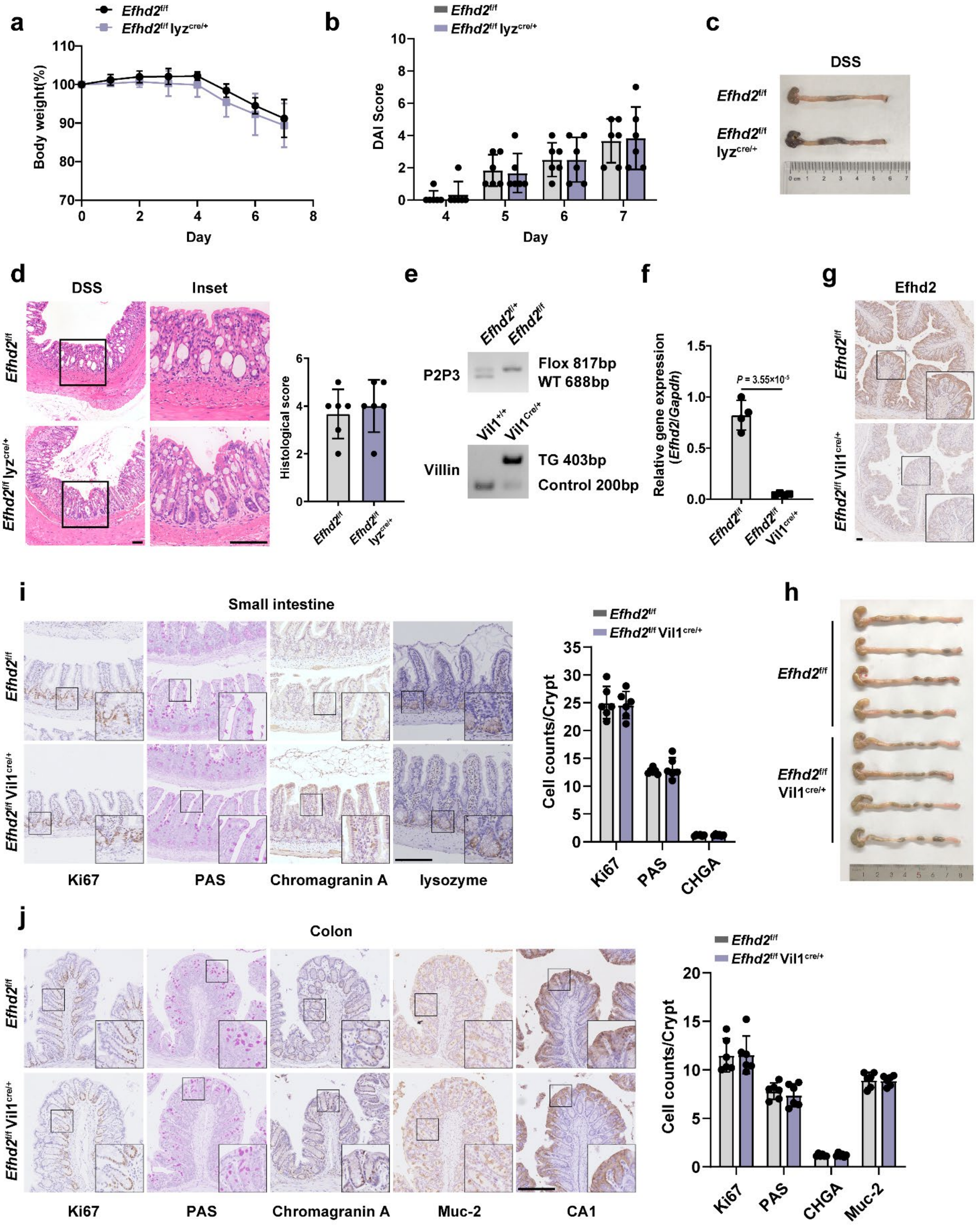

Supplementary figure 3. IEC-specific but not myeloid cell-specific ablation of *Efhd2* exacerbates colonic inflammation in response to DSS. a, b Relative

percentage of body weight change (**a**) and disease DAI score (**b**) of *Efhd2*$^{f/f}$ and *Efhd2*$^{f/f}$ Lyz$^{cre/+}$ mice administrated with 2.5% DSS for 6 days and sacrificed on day 7. **c** Representative images of colon morphology and lengths of *Efhd2*$^{f/f}$ and *Efhd2*$^{f/f}$ Lyz$^{cre/+}$ mice on day 7 after DSS administration. **d** Representative images and histologic scores of H&E staining of distal colon from *Efhd2*$^{f/f}$ and *Efhd2*$^{f/f}$ Lyz$^{cre/+}$ mice on day 7 after DSS administration. Left scale bar, 100 μm; right scale bar, 200 μm; n = 6 mice per group (**a**, **b**, **d**). **e-g** Characterization of specific deletion of *Efhd2* in IECs by genomic PCR (**e**), qPCR of isolated primary IECs (**f**) and immunostaining for Efhd2 (**g**) on mouse distal colon tissues from *Efhd2*$^{f/f}$ and *Efhd2*$^{f/f}$ Vil1$^{cre/+}$ mice. Scale bar, 100 μm. n = 4 in **f**. **h-j** Representative images of the colon morphology (**h**) and immunostaining for Ki67, PAS, Chromagranin-A, or Lysozyme in the small intestine (**i**) and for Ki67, PAS, Charomagranin-A, Muc-2, or CA1 in the colon (**j**) of *Efhd2*$^{f/f}$ and *Efhd2*$^{f/f}$ Vil1$^{cre/+}$ mice at steady-state conditions. Quantifications were shown in the histogram. n = 6. Scale bar: 200 μm. Data are representative of three independent experiments; error bars show means ± s.d. *P* values were determined by unpaired two-tailed *t*-test. For body weight curves, two-way ANOVA analysis with Sidak's multiple comparisons test. Source data are provided as a Source Data file.

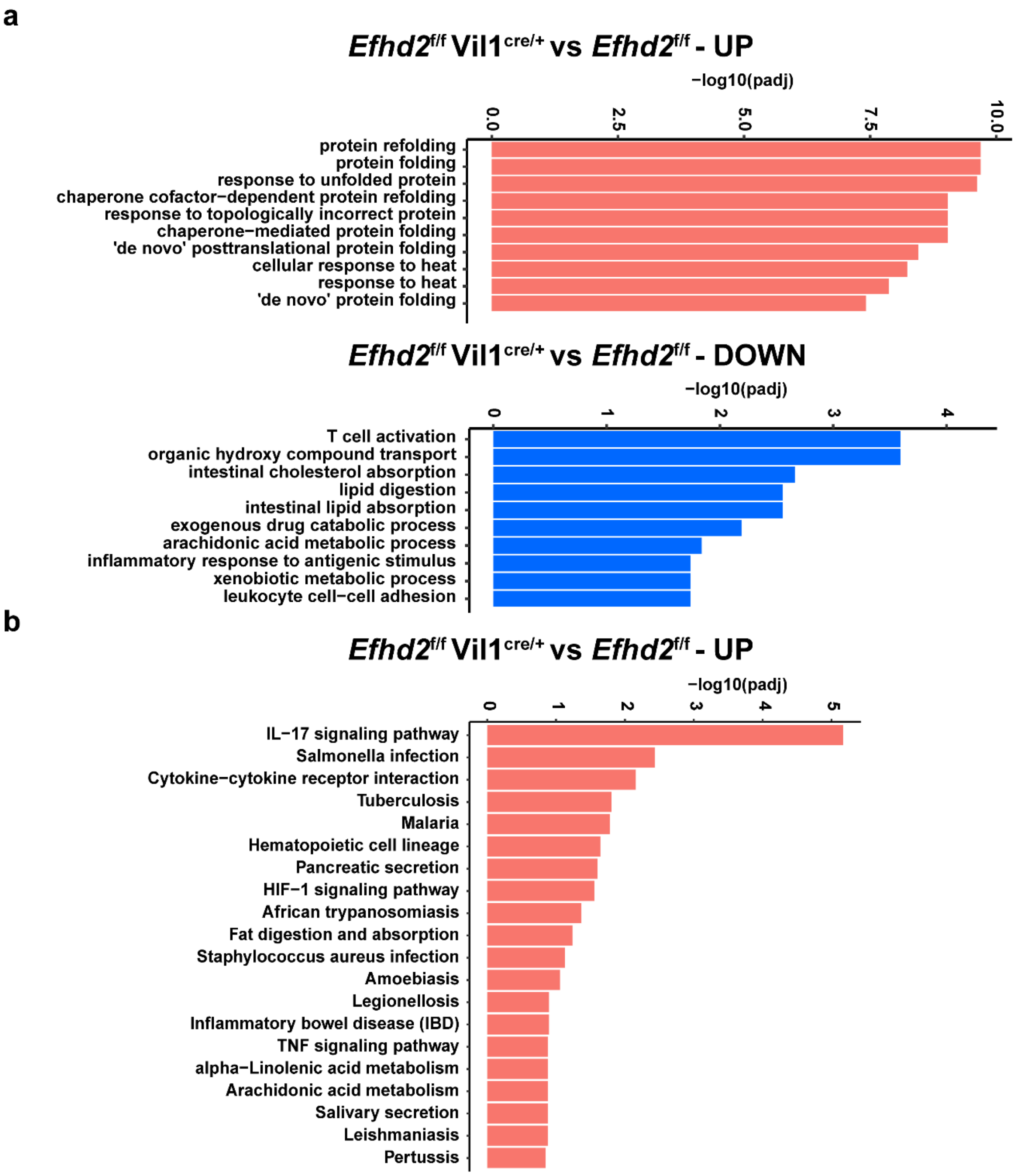

Supplementary figure 4. Transcriptional signature from isolated *Efhd2*-deficient IECs is associated with increased inflammatory pathways after DSS administration. a Gene Ontology enrichment (-log$_{10}$ padj) for biological processes from significantly up- (top) and down-regulated (bottom) transcripts in the transcriptome of isolated IECs from *Efhd2*$^{f/f}$ Vil1$^{cre/+}$ versus *Efhd2*$^{f/f}$ mice at steady-state

conditions. **b** KEGG pathway analysis ($-\log_{10}$ padj) of the up-regulated genes in the transcriptome of isolated IECs from *Efhd2*$^{f/f}$ Vil1$^{cre/+}$ versus *Efhd2*$^{f/f}$ mice after DSS administration on day 7. Source data are provided as a Source Data file.

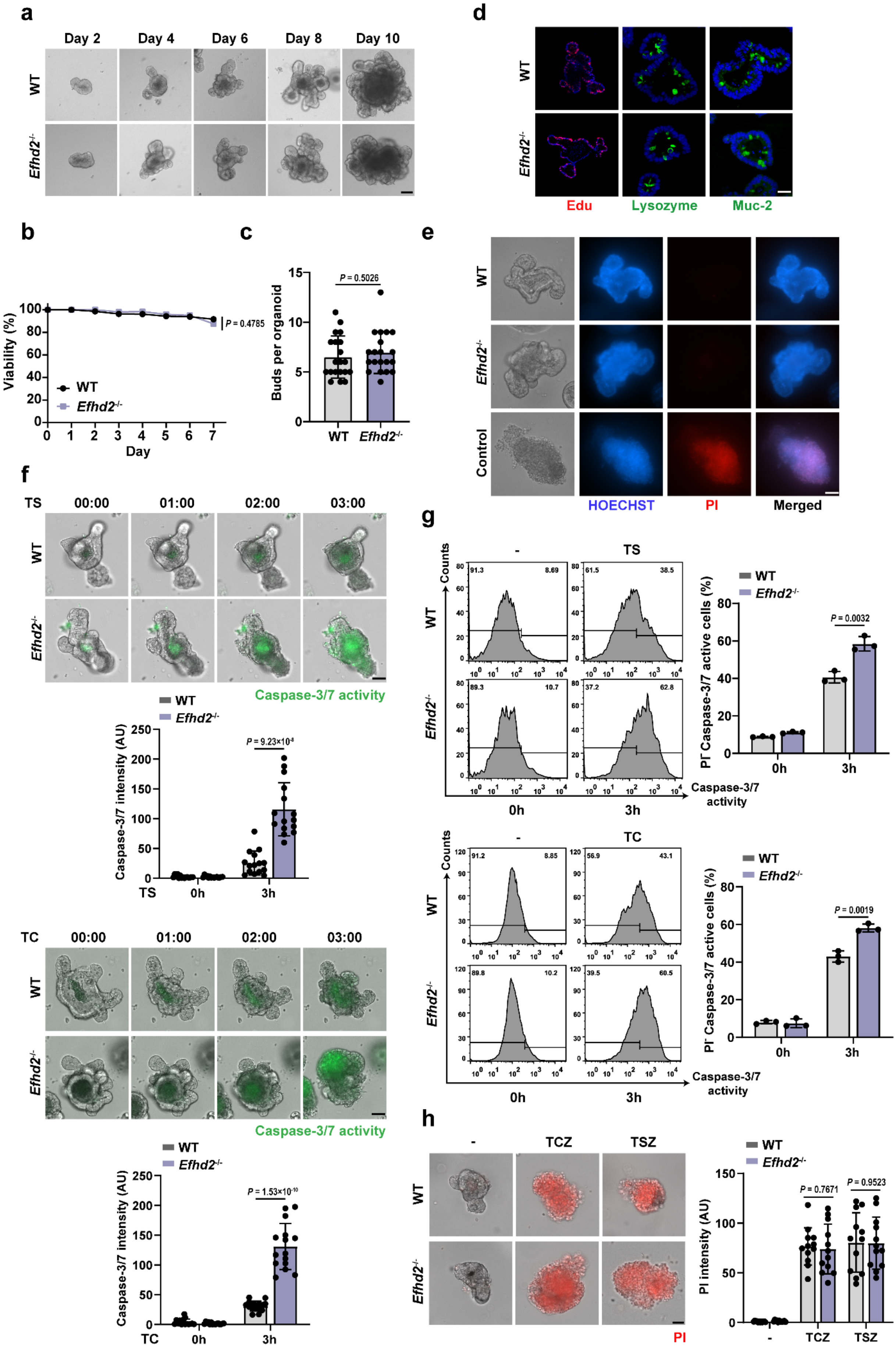
a
Day 2
Day 4
Day 6
Day 8
Day 10
WT
Efhd2-/-
b
Viability (%)
Day
P = 0.4785
WT
Efhd2-/-
c
Buds per organoid
P = 0.5026
WT
Efhd2-/-
d
WT
Efhd2-/-
Edu
Lysozyme
Muc-2
e
WT
Efhd2-/-
Control
HOECHST
PI
Merged
f
TS
00:00
01:00
02:00
03:00
Caspase-3/7 activity
Caspase-3/7 intensity (AU)
P = 9.23×10-8
0h
3h
TC
P = 1.53×10-10
g
Counts
-
TS
TC
0h
3h
Caspase-3/7 activity
PI- Caspase-3/7 active cells (%)
P = 0.0032
P = 0.0019
h
TCZ
TSZ
PI
PI intensity (AU)
P = 0.7671
P = 0.9523

Supplementary figure 5. EFHD2 protects intestinal enteroids from TNF-induced apoptosis but not necroptosis. a, **b** Representative images (**a**) and viability quantification (**b**) of WT and *Efhd2*$^{-/-}$ enteroids cultured for the indicated time. Scale bar, 50 μm. At least 50 organoids were quantified in **b**. **c** Bud number of enteroids were quantified on day 5. n = 20 enteroids each group. **d** Representative immunofluorescence images of Edu (red), lysozyme (green), Muc-2 (green), and DAPI (blue) staining of WT and *Efhd2*$^{-/-}$ enteroids on day 5. Scale bar, 50 μm. **e** Representative live cell images of WT and *Efhd2*$^{-/-}$ enteroids cultured on day 5 in the presence of PI (red) and HOECHST (blue). WT enteroids treated with TCZ for 16h as the positive control. Scale bar, 50 μm. **f** Time-series images of WT and *Efhd2*$^{-/-}$ enteroids of day 5 treated with TS or TC in the presence of a caspase-3/7 activity detection reagent (green). Digits represent hours: minutes; scale bars, 50 μm. Quantification of caspase-3/7 intensity at the indicated time was shown in the histogram. n = 15 enteroids each group. **g** Flow cytometric analysis of caspase-3/7 activity from dispersed WT and *Efhd2*$^{-/-}$ enteroids of day 5 treated with with TS or TC for 3 hours. n = 3 mice each group. **h** Representative images of WT and *Efhd2*$^{-/-}$ enteroids of day 5 treated with TCZ or TSZ for 16 hours in the presence of PI (red). Scale bar, 50 μm. Quantification of PI intensity was shown in the histogram. n = 12 enteroids each group. Data are representative of three independent experiments; error bars show means ± s.d. *P* values were determined by unpaired two-tailed *t*-test (**c**,**f**-**h**) and paired two-tailed *t*-test (**b**). Source data are provided as a Source Data file.

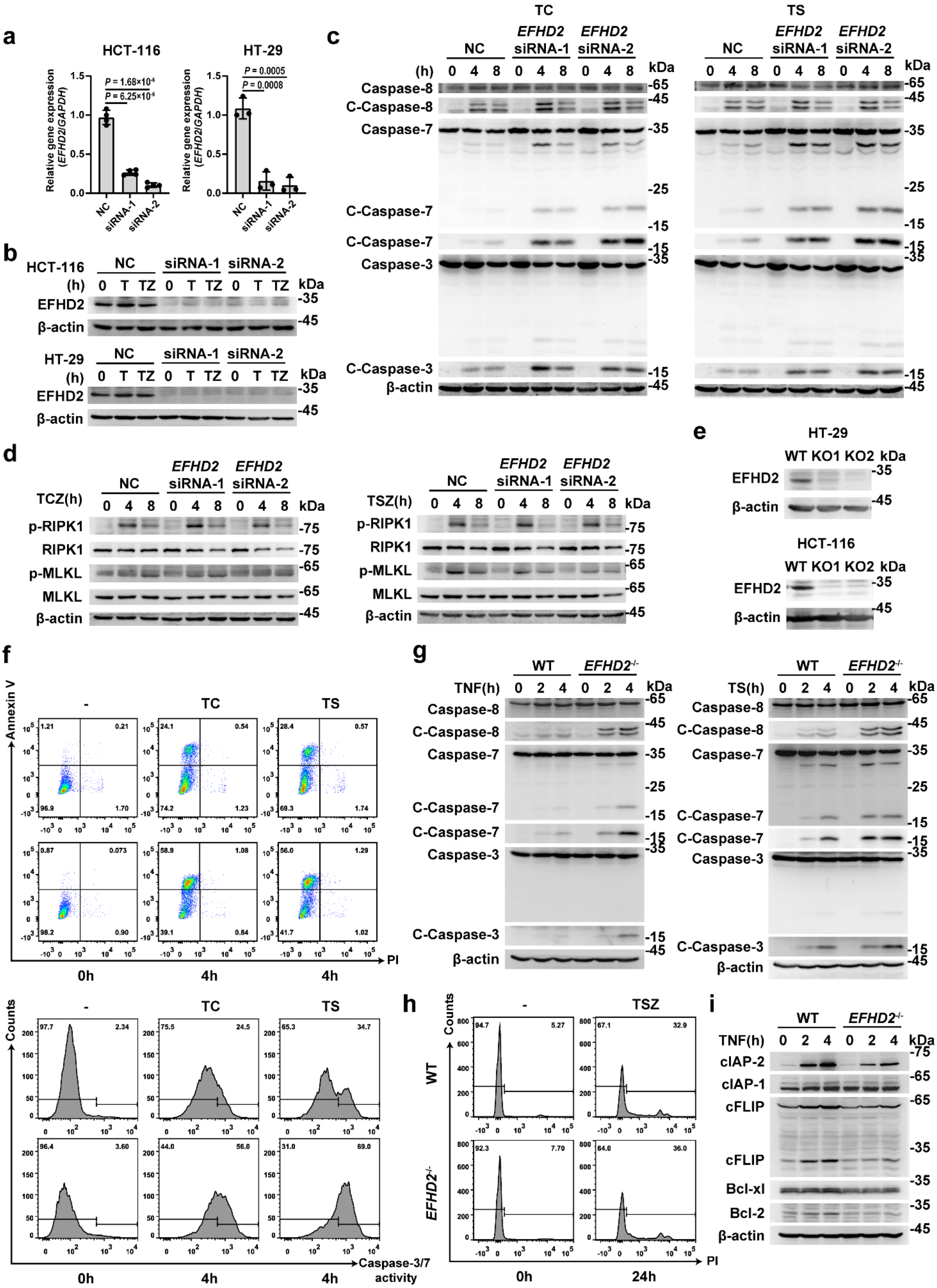
a
HCT-116
HT-29
Relative gene expression (EFHD2/GAPDH)
P = 1.68×10⁻⁶
P = 6.25×10⁻⁶
P = 0.0005
P = 0.0008
NC
siRNA-1
siRNA-2
b
HCT-116
HT-29
(h)
0 T TZ 0 T TZ 0 T TZ
EFHD2
β-actin
kDa
c
TC
TS
EFHD2 siRNA-1
EFHD2 siRNA-2
0 4 8 0 4 8 0 4 8
Caspase-8
C-Caspase-8
Caspase-7
C-Caspase-7
Caspase-3
C-Caspase-3
d
TCZ(h)
TSZ(h)
p-RIPK1
RIPK1
p-MLKL
MLKL
e
WT KO1 KO2
f
Annexin V
PI
WT
EFHD2-/-
0h
4h
Counts
Caspase-3/7 activity
g
TNF(h)
TS(h)
0 2 4 0 2 4
h
TSZ
24h
i
cIAP-2
cIAP-1
cFLIP
Bcl-xl
Bcl-2

Supplementary figure 6. EFHD2 suppresses TNF-induced apoptosis but not necroptosis in epithelial cells. a Relative mRNA levels of *EFHD2* from HCT-116 (n = 4) and HT-29 cells (n = 3) transfected with two siRNA against *EFHD2* by qPCR assay. **b** Western blot analysis for the protein level of EFHD2 in wild-type and *EFHD2* interfered HCT-116 and HT-29 cells treated with TNF alone or TNF combined with zVAD-fmk for 2h. **c**, **d** Western blot analysis of lysates from wild-type and *EFHD2* interfered HT-29 cells treated with TC and TS (**c**), or TCZ and TSZ (**d**) for the indicated time. **e** Western blot analysis for the protein level of EFHD2 in WT and $EFHD2^{-/-}$ HCT-116 and HT-29 cells. **f** Representative flow cytometric analysis of cell apoptosis and caspase-3/7 activity from WT and $EFHD2^{-/-}$ HCT-116 cells treated with TC or TS for the indicated time. **g** Western blot analysis of lysates from WT and $EFHD2^{-/-}$ HCT-116 cells treated with TNF alone or TS for the indicated time. **h** Flow cytometric analysis of PI-positive cells from WT and $EFHD2^{-/-}$ HT-29 cells treated with TSZ for the indicated time. **i** Western blot analysis for the level of NF-κB targeted genes from WT and $EFHD2^{-/-}$ HCT-116 cells treated with TNF for the indicated time. Data are representative of three independent experiments; error bars show means ± s.d. *P* values were determined by unpaired two-tailed *t*-test. Source data are provided as a Source Data file.

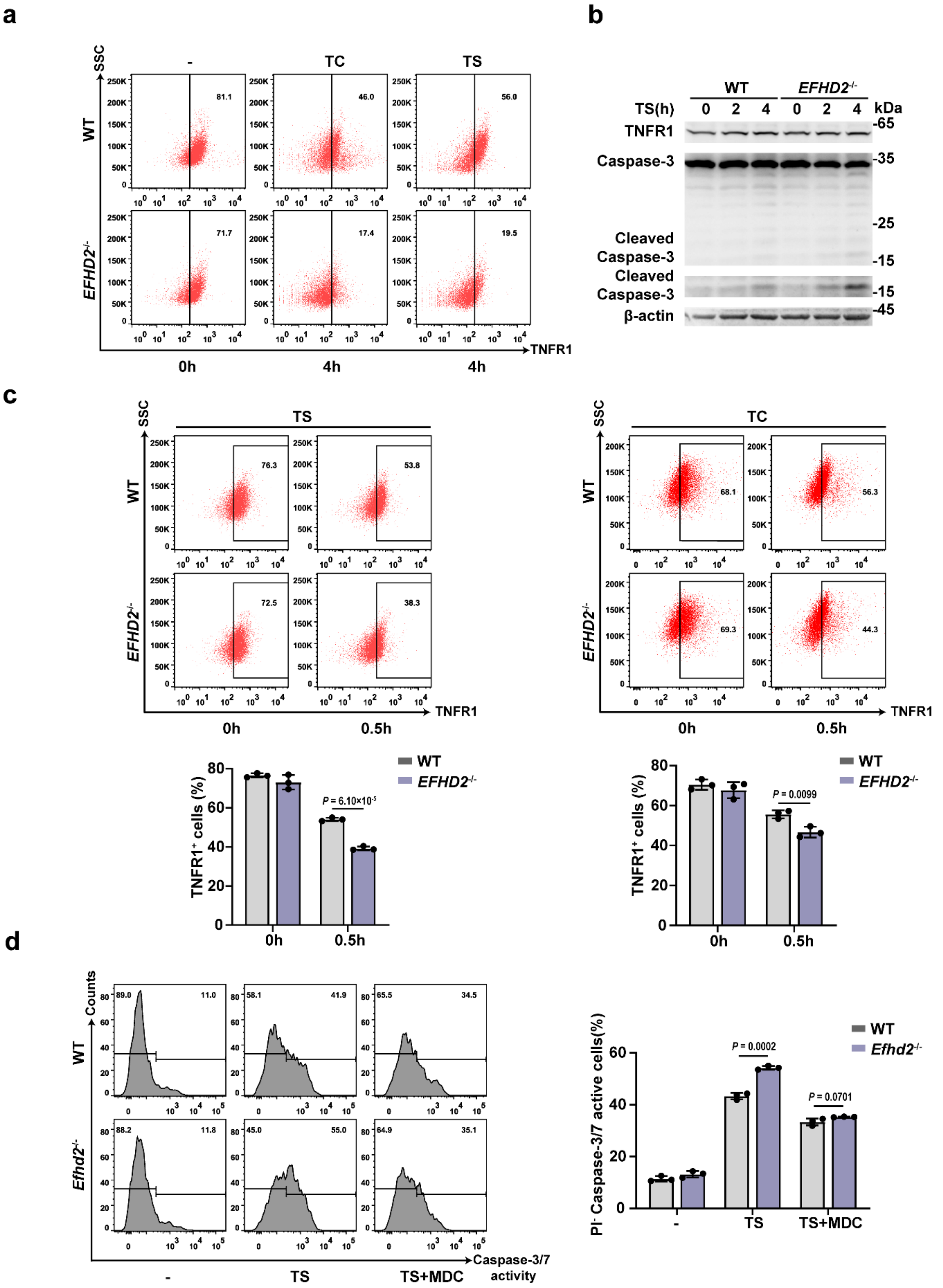

Supplementary figure 7. EFHD2 inhibits the internalization of TNFR1 in epithelial cells. a Representative flow cytometric analysis plot and proportions of cell-

surface TNFR1-positive cells from WT and *EFHD2*$^{-/-}$ HCT-116 cells treated with TC or TS for 4h. **b** Western blot analysis for the level of TNFR1 from WT and *EFHD2*$^{-/-}$ HCT-116 cells treated with TS for the indicated time. **c** Representative flow cytometric analysis plot and proportions of cell-surface TNFR1-positive cells from WT and *EFHD2*$^{-/-}$ HT-29 cells treated with TS or TC for 30 min. n = 3. **d** Flow cytometric analysis of caspase-3/7 activity from dispersed WT and *Efhd2*$^{-/-}$ enteroids treated with TS for 3 hours, without or with MDC pretreatment for 1 hour. n = 3. Data are representative of three independent experiments; error bars show means ± s.d. *P* values were determined by unpaired two-tailed *t*-test. Source data are provided as a Source Data file.

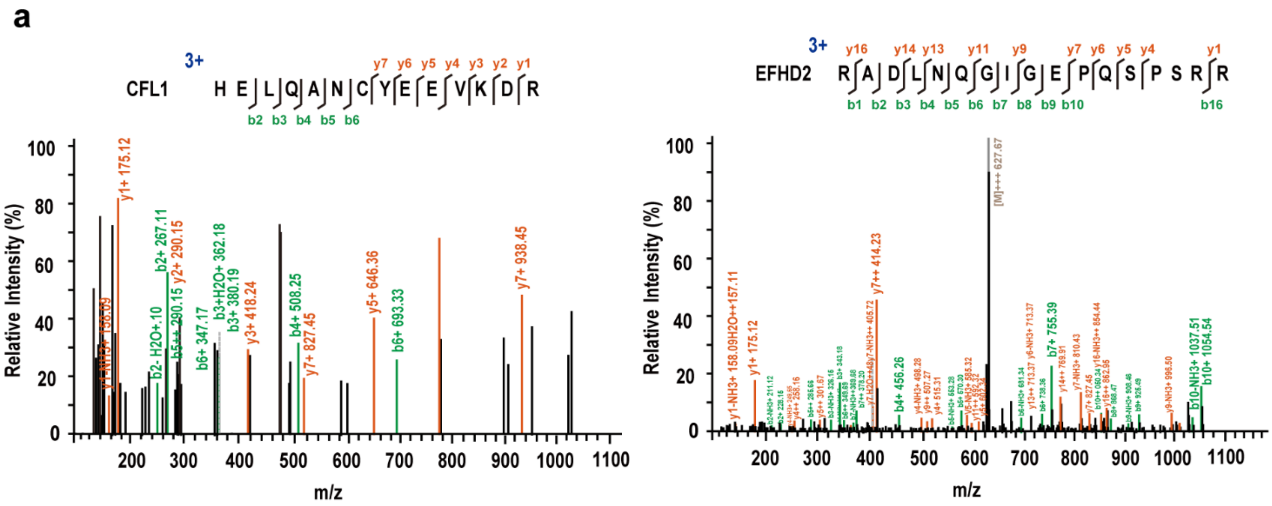

Supplementary figure 8. EFHD2 interacts with Cofilin. **a** Mass spectrometry analysis of peptides of Cofilin-1 and EFHD2. Data are representative of three independent experiments.

Supplementary Table 1 Sequences of primers for genotyping and sgRNA sequences

Primer Symbol	Primers (5'-3')
P1 for *Efhd2*$^{-/-}$ mice	ACACTGAGACCATCTTCCACATCT
P2 for *Efhd2*$^{-/-}$ mice	CATATCCGCAACTCCATGCTGTT
P3 for *Efhd2*$^{-/-}$ mice	CACTTGGGCCTCAAGAGTAT
transgene forward for *Efhd2*$^{f/f}$ Vil1$^{cre/+}$ mice	TCGATGCAACGAGTGATGAG
transgene reverse for *Efhd2*$^{f/f}$ Vil1$^{cre/+}$ mice	TCCATGAGTGAACGAACCTG
internal positive control forward for *Efhd2*$^{f/f}$ Vil1$^{cre/+}$ mice	CAAATGTTGCTTGTCTGGTG
internal positive control reverse for *Efhd2*$^{f/f}$ Vil1$^{cre/+}$ mice	GTCAGTCGAGTGCACAGTTT
wild-type for *Efhd2*$^{f/f}$ Lyz$^{cre/+}$ mice	TTACAGTCGGCCAGGCTGAC
common for *Efhd2*$^{f/f}$ Lyz$^{cre/+}$ mice	CTTGGGCTGCCAGAATTTCTC
mutation for *Efhd2*$^{f/f}$ Lyz$^{cre/+}$ mice	CCCAGAAATGCCAGATTACG
**sgRNA**	**Sequence (5'-3')**
sgRNA targeting *EFHD2* (exon 1)	GGCCAGCTCGTCCGTGGCCA
sgRNA targeting *EFHD2* (exon 1)	GCGGCGCGCAGACCTCAACC
sgRNA targeting *EFHD2* (exon 2)	GTCGATGAAGCCGTCCCGCC
sgRNA targeting *EFHD2* (exon 2)	GAGCTTCCGGGAGGTAAGCC

Supplementary Table 2 Demographics of enrolled participants

	Overall	Healthy control	UC	Remission
**Count**	29	9	10	10
**Male**	14	5	6	3
**Female**	15	4	4	7
**Median age**	52	60	50.5	45.5
**Min age**	24	38	30	24
**Max age**	73	66	69	73
**Region of sampling**				
Ascending colon	3	2	1	0
Transverse colon	4	0	1	3
Descending colon	3	2	0	1
Sigmoid colon	17	4	8	5
Rectum	2	1	0	1

Median age: 52
Mean age: 50.55

Supplementary Table 3 Demographics of UC patients with infliximab treatment

	Infliximab responder	Infliximab non-responder
**Number of patients (*n*)**	5	6
**Sex, women, *n* (%)**	3(60%)	3(50%)
**Age (yrs), mean (±SEM)**	37.20 ± 1.685	38.00 ± 3.856
**Region of sampling**		
Cecum	1	0
Ascending colon	1	0
Transverse colon	0	1
Descending colon	1	1
Sigmoid colon	2	4

Supplementary Table 4 EFHD2-interacting proteins identified by LC-MS analysis in HCT-116 cells

0min				
**Accession**	**Gene Name**	**Score**	**Matches**	**Coverage**
A0A024QZ77	EFHD2	11132	532(345)	66%
A0A075B6S2	IGKV2D-29	812	33(22)	19%
A0A5C2FZI3		748	16(15)	20%
H6VRF8	KRT1	648	44(17)	39%
H6VRG2	KRT1	639	44(17)	39%
P13645	KRT10	620	50(18)	42%
P35908	KRT2	568	32(16)	29%
Q71U36	TUBA1A	370	25(11)	38%
A0A6Q8PFK8	HSPB1	191	9(5)	45%
A0A5C2G8A6		176	6(5)	8%
P35527	KRT9	171	29(10)	29%
A0A384ME17	TUFM	167	9(5)	21%
A0A075B6Z2	TRAJ56	161	17(15)	38%
Q6I9V5	SLC25A6	151	9(5)	18%
A0A0S2Z4Y5	DPM1	141	11(5)	28%
B3KML9		125	16(5)	31%
P08779	KRT16	114	21(5)	22%
G3V1A4	CFL1	113	6(4)	24%
A0A5C2GC48		112	17(9)	20%
P27348	YWHAQ	105	3(3)	9%
Q9NS69	TOMM22	99	3(2)	16%
A0A075B6R9	IGKV2D-24	99	15(9)	13%
Q8TBT6		98	9(2)	18%
A0A0S2Z382	SLC25A10	92	18(6)	31%
P02533	KRT14	89	17(4)	19%
A0A804GS07	ACTG1	87	7(3)	16%
A0A024RBE8	SLC25A3	84	13(3)	12%
P05787	KRT8	84	11(3)	15%
A0A5C2GAH8		82	7(4)	11%
B4DZG7	ARL1	79	3(2)	10%
V9HW22	HEL-S-72p	78	7(3)	14%
K7EK00	FAM210A	77	4(1)	12%
A0A0A0MRQ5	PRDX1	71	4(2)	34%
Q9UNL2	SSR3	68	1(1)	7%
A0A024RD80	HSP90AB1	67	10(1)	12%
A0A384MTW7		65	1(1)	7%
I3L1P8	SLC25A11	65	2(2)	8%
P08727	KRT19	63	17(3)	27%
O95881	TXNDC12	62	2(2)	13%
D9HTE9	SLC25A1	58	5(2)	16%
Q0QET7	GAPDH	55	2(1)	6%

A0A024R5Z7	ANXA2	49	1(1)	2%
P18085	ARF4	39	6(2)	21%
E9PL09	RPS3	39	5(1)	18%
Q96DM1	PGBD4	37	3(1)	4%
J3KS45	TMCO1	37	1(1)	10%
L0R5A1	CSF2RB	36	6(3)	7%
A0A286YEY4	IGHG2	36	1(1)	2%
A0A2R8Y6V5	CCSER2	35	3(1)	7%
A0A2R8Y653	UCK2	34	4(0)	18%
A0A087WVQ9	EEF1A1	32	9(1)	9%
A0A2X0TVV1	ARHGEF17	30	7(1)	0%
Q8WUK0	PTPMT1	29	4(1)	19%
A8K2M8		29	3(0)	3%
E9PLX7	RPL27A	29	7(1)	18%
Q9NPL8	TIMMDC1	28	3(1)	12%
Q9Y6C9	MTCH2	28	1(1)	5%
A0A140T907	FLOT1	25	2(0)	21%
B2R4I8		25	1(0)	7%
J3QRS3	MYL12A	23	1(0)	5%
A0A087WWT3	ALB	23	3(0)	3%
J3QSB4	RPL13	23	3(0)	23%
A0A024R8P8	RPL38	21	4(0)	35%
P62280	RPS11	18	4(0)	29%
Q8IZ22	MKKS	17	2(0)	2%
A0A087WW66	PSMD1	16	9(0)	2%

**TNF 15min**				
**Accession**	**Gene Name**	**Score**	**Matches**	**Coverage**
A0A024QZ77	EFHD2	12046	546(359)	70%
H6VRF8	KRT1	909	66(25)	44%
A0A5C2GLS3		835	40(25)	43%
A0A1B0GVI3	KRT10	457	47(18)	24%
P35527	KRT9	382	41(13)	39%
P35908	KRT2	361	31(8)	27%
P81605	DCD	233	8(5)	22%
P04259	KRT6B	220	19(8)	16%
Q9BQE3	TUBA1C	220	17(6)	33%
A0A384ME17	TUFM	170	6(3)	14%
E9PK25	CFL1	164	8(4)	24%
A0A075B6R9	IGKV2D-24	147	16(11)	13%
A0A075B6Z2	TRAJ56	132	16(13)	38%
A0A0S2Z4Y5	DPM1	109	13(3)	36%
A0A5C2GC48		107	21(10)	20%
A0A5C2G8A6		105	6(4)	8%
P02533	KRT14	105	18(4)	19%
Q6I9V5	SLC25A6	100	8(4)	17%
A0A7S5EVE0		98	7(4)	18%
A0A0S2Z382	SLC25A10	91	12(4)	26%

A0A6Q8PFK8	HSPB1	86	7(2)	31%
P18085	ARF4	70	6(2)	15%
Q9UNL2	SSR3	68	1(1)	7%
A0A384NYT8		67	11(4)	20%
Q8TBT6		65	7(1)	13%
Q9NS69	TOMM22	64	4(1)	8%
A0A384MTW7		64	1(1)	7%
A0A0A0MSI0	PRDX1	59	5(1)	23%
K7EK00	FAM210A	58	1(1)	5%
E9PKE3	HSPA8	57	5(1)	10%
P05787	KRT8	53	9(1)	8%
Q9Y5M8	SRPRB	51	1(1)	5%
P46783	RPS10	48	3(2)	23%
L0R5A1	CSF2RB	48	4(3)	7%
A0A024RBE8	SLC25A3	45	6(1)	8%
A8K1K8	C18orf55	42	3(1)	10%
Q59FC6		40	5(1)	7%
Q02413	DSG1	38	2(1)	3%
A0A6Q8PFE4	ACTB	33	6(2)	20%
Q96DM1	PGBD4	30	1(1)	1%
D9HTE9	SLC25A1	28	4(1)	14%
A0A024R5Z7	ANXA2	28	2(0)	7%
Q8TBC8	C21orf57	28	1(0)	13%
Q96RW7	HMCN1	28	2(1)	0%
A0A286YEY4	IGHG2	24	2(0)	2%
Q7Z3K9	DKFZp781L0540	19	3(0)	4%
A0A140VK00		18	3(0)	7%
I3L1P8	SLC25A11	18	3(0)	9%
L0R5D5	TMEM70	17	1(0)	9%
Q8N584	TTC39C	16	1(0)	2%
A0A2R8Y811	RPS14	16	1(0)	8%
I3L3I7	PLD2	15	3(0)	5%

Supplementary Table 5 Sequences of primers for qPCR and siRNA sequences

Gene Symbol	Primers (5’-3’)	
	**Forward**	**Reverse**
Il1b	GCAACTGTTCCTGAACTCAACT	ATCTTTTGGGGTCCGTCAACT
Il1a	CGAAGACTACAGTTCTGCCATT	GACGTTTCAGAGGTTCTCAGAG
Il6	TAGTCCTTCCTACCCCAATTTC C	TTGGTCCTTAGCCACTCCTTC
Efhd2	CAGGAGGTGGACGAGGATTTC	AAAGCGGCTGGACACGTTGAT
Gapdh	AGGTCGGTGTGAACGGATTTG	TGTAGACCATGTAGTTGAGGTC A
EFHD2	TTCCTCCTGATCTTCCGCAAGG C	CTACTTAAAGGTGGACTGCAGC
GAPDH	ACAACTTTGGTATCGTGGAAGG	GCCATCACGCCACAGTTTC

**siRNA**	**Sequence (5’-3’)**
siRNA targeting *EFHD2* (sense 1)	ACACCGAGTTCAAGGAGTT
siRNA targeting *EFHD2* (sense 2)	GCTTCGAGGAGGAGATCAA
