# Supplementary material for: EFHD2 suppresses intestinal inflammation by blocking intestinal epithelial cell TNFR1 internalization and cell death
Source: Nat Commun. 2024 Feb 12;15:1282. doi: 10.1038/s41467-024-45539-x (PMC10861516; doi:10.1038/s41467-024-45539-x)
Supplement: Supplementary file 3 — Reporting Summary [file 41467_2024_45539_MOESM3_ESM.pdf]

nature portfolio

Corresponding author(s): Xuetao Cao

Last updated by author(s): Jan 16, 2024

# Reporting Summary

Nature Portfolio wishes to improve the reproducibility of the work that we publish. This form provides structure for consistency and transparency in reporting. For further information on Nature Portfolio policies, see our Editorial Policies and the Editorial Policy Checklist.

## Statistics

For all statistical analyses, confirm that the following items are present in the figure legend, table legend, main text, or Methods section.

| n/a | Confirmed | |
|---|---|---|
| ☐ | ☒ | The exact sample size (*n*) for each experimental group/condition, given as a discrete number and unit of measurement |
| ☐ | ☒ | A statement on whether measurements were taken from distinct samples or whether the same sample was measured repeatedly |
| ☐ | ☒ | The statistical test(s) used AND whether they are one- or two-sided<br>*Only common tests should be described solely by name; describe more complex techniques in the Methods section.* |
| ☒ | ☐ | A description of all covariates tested |
| ☐ | ☒ | A description of any assumptions or corrections, such as tests of normality and adjustment for multiple comparisons |
| ☐ | ☒ | A full description of the statistical parameters including central tendency (e.g. means) or other basic estimates (e.g. regression coefficient) AND variation (e.g. standard deviation) or associated estimates of uncertainty (e.g. confidence intervals) |
| ☐ | ☒ | For null hypothesis testing, the test statistic (e.g. *F*, *t*, *r*) with confidence intervals, effect sizes, degrees of freedom and *P* value noted<br>*Give P values as exact values whenever suitable.* |
| ☒ | ☐ | For Bayesian analysis, information on the choice of priors and Markov chain Monte Carlo settings |
| ☒ | ☐ | For hierarchical and complex designs, identification of the appropriate level for tests and full reporting of outcomes |
| ☒ | ☐ | Estimates of effect sizes (e.g. Cohen's *d*, Pearson's *r*), indicating how they were calculated |

*Our web collection on statistics for biologists contains articles on many of the points above.*

## Software and code

Policy information about availability of computer code

Data collection

1. FACS experiments was performed by BD LSRFortessa flow cytometer (BD Biosciences).
2. Live imaging of organoid cell death was acquired using the ZEISS Celldiscoverer 7 imaging system (ZEISS).
3. Real-time PCR analysis was performed by QuantStudio 7 Flex Real-Time PCR System (Thermo Fisher Scientific)
4. Transcriptome sequencing was performed using the Illumina NovaSeq 6000 platform (Novegene).
5. SynergyH1 automatic microplate reader (Biotek).
6. Immunohistological images were acquired using Aperio XT (LEICA) and NanoZoomer S360 (Hamamastu).
7. Immunofluorescence images were acquired using Leica TCS SP8 gSTED 3X (Leica) and Pannoramic MIDI (3DHISTECH).
8. Mass cytometry by time-of-flight (CyTOF) was performed using Halios (Fluidigm).

Data analysis

1. Differential expression analysis from transcriptome sequencing was analyzed by the DESeq2 R package. GO and KEGG enrichment analysis were implemented by the clusterProfiler R package.
2. Statistical analysis was performed using the GraphPad Prism 8.0.
3. All mass cytometry files were analyzed using Cytobank software and R cytofkit package.
4. Flow cytometry data were analyzed by Flowjo_V10.
5. Quantifications of western-blots were analyzed by ImageJ (V 1.8.0).

For manuscripts utilizing custom algorithms or software that are central to the research but not yet described in published literature, software must be made available to editors and reviewers. We strongly encourage code deposition in a community repository (e.g. GitHub). See the Nature Portfolio guidelines for submitting code & software for further information.

## Data

Policy information about availability of data

All manuscripts must include a data availability statement. This statement should provide the following information, where applicable:

- Accession codes, unique identifiers, or web links for publicly available datasets
- A description of any restrictions on data availability
- For clinical datasets or third party data, please ensure that the statement adheres to our policy

The RNA high throughput sequencing data of this study is deposited in the NCBI GEO dataset under accession code GSE224768 (https://www.ncbi.nlm.nih.gov/geo/query/acc.cgi?acc=GSE224768). The public single-cell RNA sequencing dataset in the re-analysis for EFHD2 expression levels in multiple cell types from normal human colon tissue is deposited in the NCBI GEO dataset under accession code GSE116222 (https://www.ncbi.nlm.nih.gov/geo/query/acc.cgi?acc=GSE116222). The mass spectrometry data reported in this study is deposited in the ProteomeXchange with the dataset identifier PXD048587 (https://proteomecentral.proteomexchange.org/cgi/GetDataset?ID=PXD048587). All other study data are included in the article and/or Supplementary Information. Any additional information is available upon request to the corresponding author (Xuetao Cao, caoxt@immunol.org). Source data are provided with this paper.

## Research involving human participants, their data, or biological material

Policy information about studies with human participants or human data. See also policy information about sex, gender (identity/presentation), and sexual orientation and race, ethnicity and racism.

| | |
|---|---|
| Reporting on sex and gender | The gender/sex information was listed in Supplementary Table 1 and 2. |
| Reporting on race, ethnicity, or other socially relevant groupings | All patients we recruited are Chinese people. |
| Population characteristics | The clinical characteristics of  enrolled patients were all listed in Supplementary Table 1 and 2. |
| Recruitment | The human intestinal biopsy samples for this study were obtained from the colons of UC patients at the active or remission stage (n = 10 for each group) and healthy controls (n = 9 for each group). They were collected through colonoscopic biopsy by Peking Union Medical College Hospital. The human intestinal colonoscopic biopsy samples from UC patients that were responsive and non-responsive to infliximab treatment were collected by Peking Union Medical College Hospital. |
| Ethics oversight | The Ethics Review Board of Peking Union Medical College Hospital approved the study design and experimental procedures, the use of pathological specimens and the review of pertinent patient records (ZS-2178). Informed consent was obtained from all subjects. |

Note that full information on the approval of the study protocol must also be provided in the manuscript.

# Field-specific reporting

Please select the one below that is the best fit for your research. If you are not sure, read the appropriate sections before making your selection.

[x] Life sciences  [ ] Behavioural & social sciences  [ ] Ecological, evolutionary & environmental sciences

For a reference copy of the document with all sections, see nature.com/documents/nr-reporting-summary-flat.pdf

# Life sciences study design

All studies must disclose on these points even when the disclosure is negative.

| | |
|---|---|
| Sample size | No statistical methods were employed to predetermine the sample size. Sample sizes were determined in accordance with standard practices. For in vivo experiments, group sizes were selected empirically, based on prior knowledge of intra-group variation associated with DSS- or TNF-induced colitis using similar approaches. We usually use at least 5 mice per group. Likewise, in vitro experiments employed group sizes based on prior knowledge of variation, with three biological replicates utilized (https://doi.org/10.1038/s41556-021-00708-8; https://doi.org/10.1038/s41419-018-1275-5; https://doi.org/10.1084/jem.20170558). The number of independent experiments and biological replicates was indicated in each figure legend. |
| Data exclusions | After check the data quality , all data were included. |
| Replication | All the reported experiments were reproducible. Data reproducibility was confirmed by independent experiments. All figure legends included repeat times. |
| Randomization | Experiment mice were randomly allocated into different experimental and control groups. Other experiments were not relevant as the results are qualitative. |
| Blinding | Investigators were blinded during data collection (weight and DAI score) for all the mice experiments. The quantifications for immunofluoresecene and western-blot assays were performed blindly. For histological analyses, slides were blinded prior to scoring. For other |

experiments, blinding is not relevant to our study as the investigators need to be aware of the genotypes of the cell lines and mouse strains. The phenotype change is beyond what can be affect by human bias.

# Behavioural & social sciences study design

All studies must disclose on these points even when the disclosure is negative.

| | |
|---|---|
| Study description | *Briefly describe the study type including whether data are quantitative, qualitative, or mixed-methods (e.g. qualitative cross-sectional, quantitative experimental, mixed-methods case study).* |
| Research sample | *State the research sample (e.g. Harvard university undergraduates, villagers in rural India) and provide relevant demographic information (e.g. age, sex) and indicate whether the sample is representative. Provide a rationale for the study sample chosen. For studies involving existing datasets, please describe the dataset and source.* |
| Sampling strategy | *Describe the sampling procedure (e.g. random, snowball, stratified, convenience). Describe the statistical methods that were used to predetermine sample size OR if no sample-size calculation was performed, describe how sample sizes were chosen and provide a rationale for why these sample sizes are sufficient. For qualitative data, please indicate whether data saturation was considered, and what criteria were used to decide that no further sampling was needed.* |
| Data collection | *Provide details about the data collection procedure, including the instruments or devices used to record the data (e.g. pen and paper, computer, eye tracker, video or audio equipment) whether anyone was present besides the participant(s) and the researcher, and whether the researcher was blind to experimental condition and/or the study hypothesis during data collection.* |
| Timing | *Indicate the start and stop dates of data collection. If there is a gap between collection periods, state the dates for each sample cohort.* |
| Data exclusions | *If no data were excluded from the analyses, state so OR if data were excluded, provide the exact number of exclusions and the rationale behind them, indicating whether exclusion criteria were pre-established.* |
| Non-participation | *State how many participants dropped out/declined participation and the reason(s) given OR provide response rate OR state that no participants dropped out/declined participation.* |
| Randomization | *If participants were not allocated into experimental groups, state so OR describe how participants were allocated to groups, and if allocation was not random, describe how covariates were controlled.* |

# Ecological, evolutionary & environmental sciences study design

All studies must disclose on these points even when the disclosure is negative.

| | |
|---|---|
| Study description | *Briefly describe the study. For quantitative data include treatment factors and interactions, design structure (e.g. factorial, nested, hierarchical), nature and number of experimental units and replicates.* |
| Research sample | *Describe the research sample (e.g. a group of tagged Passer domesticus, all Stenocereus thurberi within Organ Pipe Cactus National Monument), and provide a rationale for the sample choice. When relevant, describe the organism taxa, source, sex, age range and any manipulations. State what population the sample is meant to represent when applicable. For studies involving existing datasets, describe the data and its source.* |
| Sampling strategy | *Note the sampling procedure. Describe the statistical methods that were used to predetermine sample size OR if no sample-size calculation was performed, describe how sample sizes were chosen and provide a rationale for why these sample sizes are sufficient.* |
| Data collection | *Describe the data collection procedure, including who recorded the data and how.* |
| Timing and spatial scale | *Indicate the start and stop dates of data collection, noting the frequency and periodicity of sampling and providing a rationale for these choices. If there is a gap between collection periods, state the dates for each sample cohort. Specify the spatial scale from which the data are taken* |
| Data exclusions | *If no data were excluded from the analyses, state so OR if data were excluded, describe the exclusions and the rationale behind them, indicating whether exclusion criteria were pre-established.* |
| Reproducibility | *Describe the measures taken to verify the reproducibility of experimental findings. For each experiment, note whether any attempts to repeat the experiment failed OR state that all attempts to repeat the experiment were successful.* |
| Randomization | *Describe how samples/organisms/participants were allocated into groups. If allocation was not random, describe how covariates were controlled. If this is not relevant to your study, explain why.* |
| Blinding | *Describe the extent of blinding used during data acquisition and analysis. If blinding was not possible, describe why OR explain why blinding was not relevant to your study.* |

Did the study involve field work? ☐ Yes ☐ No

## Field work, collection and transport

| | |
|---|---|
| Field conditions | *Describe the study conditions for field work, providing relevant parameters (e.g. temperature, rainfall).* |
| Location | *State the location of the sampling or experiment, providing relevant parameters (e.g. latitude and longitude, elevation, water depth).* |
| Access & import/export | *Describe the efforts you have made to access habitats and to collect and import/export your samples in a responsible manner and in compliance with local, national and international laws, noting any permits that were obtained (give the name of the issuing authority, the date of issue, and any identifying information).* |
| Disturbance | *Describe any disturbance caused by the study and how it was minimized.* |

# Reporting for specific materials, systems and methods

We require information from authors about some types of materials, experimental systems and methods used in many studies. Here, indicate whether each material, system or method listed is relevant to your study. If you are not sure if a list item applies to your research, read the appropriate section before selecting a response.

### Materials & experimental systems

| n/a | Involved in the study | |
|---|---|---|
| ☐ | ☒ | Antibodies |
| ☐ | ☒ | Eukaryotic cell lines |
| ☒ | ☐ | Palaeontology and archaeology |
| ☐ | ☒ | Animals and other organisms |
| ☒ | ☐ | Clinical data |
| ☒ | ☐ | Dual use research of concern |
| ☒ | ☐ | Plants |

### Methods

| n/a | Involved in the study | |
|---|---|---|
| ☒ | ☐ | ChIP-seq |
| ☐ | ☒ | Flow cytometry |
| ☒ | ☐ | MRI-based neuroimaging |

## Antibodies

Antibodies used

Anti-EFHD2 antibody produced in rabbit, Sigma Aldrich, HPA048961
Anti-EFHD2/SWS1 antibody, Abcam, ab24368
Anti-Chromogranin A antibody, Abcam, ab15160
Anti-Lysozyme antibody [EPR2994(2)], Abcam, ab108508
Anti -MUC2 Rabbit pAb, Servicebio, GB11344-100
Anti -Ki67 Mouse mAb, Servicebio, GB121141-100
Anti-Carbonic Anhydrase 1/CA1 antibody [EPR23232-286], Abcam, ab267475
TNF-R1 antibody (H-5), Santa Cruz, sc-8436
EEA1 (C45B10) Rabbit mAb, Cell Signaling Technology, #3288
Goat anti-Mouse IgG (H+L) Cross-Adsorbed Secondary Antibody, Alexa Fluor™ 594, Invitrogen, A-11005
Goat anti-Rabbit IgG (H+L) Cross-Adsorbed Secondary Antibody, Alexa Fluor™ 488, Invitrogen, A-11008
Cleaved Caspase-3 (Asp175) Antibody, Cell Signaling Technology, #9661
Cleaved Caspase-8 (Asp387) (D5B2) XP® Rabbit mAb, Cell Signaling Technology, #8592
Phospho-MLKL (Ser345) (D6E3G) Rabbit mAb, , #37333
Anti -E-cadherin Mouse mAb, Servicebio, GB12082-100
Caspase-7 Antibody, Cell Signaling Technology, #9492
Cleaved Caspase-8 (Asp374) (18C8) Rabbit mAb, Cell Signaling Technology, #9496
Caspase-8 (1C12) Mouse mAb, Cell Signaling Technology, #9746
Caspase-3 Antibody, Cell Signaling Technology, #9662
MLKL (D2I6N) Rabbit mAb, Cell Signaling Technology, #14993
MLKL (D6W1K) Rabbit mAb, Cell Signaling Technology, #37705
Phospho-MLKL (Ser358) (D6H3V) Rabbit mAb, Cell Signaling Technology, #91689
RIP (D94C12) XP® Rabbit mAb, Cell Signaling Technology, #3493
Phospho-RIP (Ser166) (D1L3S) Rabbit mAb, Cell Signaling Technology, #65746
Phospho-RIP (Ser166) Antibody, Cell Signaling Technology, #31122
Phospho-IKKα/β (Ser176/180) (16A6) Rabbit mAb, Cell Signaling Technology, #2697
IKKα Antibody, Cell Signaling Technology, #2682
IKKβ (D30C6) Rabbit mAb, Cell Signaling Technology, #8943
Phospho-NF-κB p65 (Ser536) (93H1) Rabbit mAb, Cell Signaling Technology, #3033
NF-κB p65 (D14E12) XP® Rabbit mAb, Cell Signaling Technology, #8242
TRADD (7G8) Rabbit mAb, Cell Signaling Technology, #3684
c-IAP1 (D5G9) Rabbit mAb, Cell Signaling Technology, #7065

c-IAP2 (58C7) Rabbit mAb, Cell Signaling Technology, #3130
TRAF2 (C192) Antibody, Cell Signaling Technology, #4724
Rab5 (C8B1) Rabbit mAb, Cell Signaling Technology, #3547
Cofilin (D3F9) XP® Rabbit mAb, Cell Signaling Technology, #5175
Phospho-Cofilin (Ser3) (77G2) Rabbit mAb, Cell Signaling Technology, #3313
Na+/K+-ATPase antibody (H-3), Santa Cruz, sc-48345
Anti-β-Actin mAb, MBL, M177-3
EFHD2, GeneTex, GTX108080
Monoclonal ANTI-FLAG® M2 antibody produced in mouse, Sigma Aldrich, F1804
TNFR1 Polyclonal antibody, Proteintech, 21574-1-AP
FLIP (D5J1E) Rabbit mAb, Cell Signaling Technology, #56343
Bcl-xL (54H6) Rabbit mAb, Cell Signaling Technology, #2764
Bcl-2 (D17C4) Rabbit mAb, Cell Signaling Technology, #3498

Validation

All antibodies were obtained commercially and were tested and validated by the respective company. All antibodies had validation statement provided on the website of the manufacturer.
Anti-EFHD2 antibody produced in rabbit, https://www.sigmaaldrich.cn/CN/zh/product/sigma/hpa048961
Anti-EFHD2/SWS1 antibody, https://www.abcam.cn/products/primary-antibodies/efhd2sws1-antibody-ab24368.html
Anti-Chromogranin A antibody, https://www.abcam.cn/products/primary-antibodies/chromogranin-a-antibody-ab15160.html
Anti-Lysozyme antibody [EPR2994(2)], https://www.abcam.cn/products/primary-antibodies/lysozyme-antibody-epr29942-ab108508.html
Anti -MUC2 Rabbit pAb, https://www.servicebio.cn/goodsdetail?id=1173
Anti -Ki67 Mouse mAb, https://www.servicebio.cn/goodsdetail?id=6801
Anti-Carbonic Anhydrase 1/CA1 antibody [EPR23232-286], https://www.abcam.cn/products/primary-antibodies/carbonic-anhydrase-1ca1-antibody-epr23232-286-ab267475.html
TNF-R1 antibody (H-5), https://www.scbt.com/zh/p/tnf-r1-antibody-h-5?requestFrom=search
EEA1 (C45B10) Rabbit mAb, https://www.cellsignal.cn/products/primary-antibodies/eea1-c45b10-rabbit-mab/3288
Cleaved Caspase-3 (Asp175) Antibody, https://www.cellsignal.cn/products/primary-antibodies/cleaved-caspase-3-asp175-antibody/9661
Cleaved Caspase-8 (Asp387) (D5B2) XP® Rabbit mAb, https://www.cellsignal.cn/products/primary-antibodies/cleaved-caspase-8-asp387-d5b2-xp-rabbit-mab/8592
Phospho-MLKL (Ser345) (D6E3G) Rabbit mAb, https://www.cellsignal.cn/products/primary-antibodies/phospho-mlkl-ser345-d6e3g-rabbit-mab/37333
Anti -E-cadherin Mouse mAb, https://www.servicebio.cn/goodsdetail?id=597
Goat anti-Mouse IgG (H+L) Cross-Adsorbed Secondary Antibody, Alexa Fluor™ 594, https://www.thermofisher.cn/cn/zh/antibody/product/Goat-anti-Mouse-IgG-H-L-Cross-Adsorbed-Secondary-Antibody-Polyclonal/A-11005
Goat anti-Rabbit IgG (H+L) Cross-Adsorbed Secondary Antibody, Alexa Fluor™ 488, https://www.thermofisher.cn/cn/zh/antibody/product/Goat-anti-Rabbit-IgG-H-L-Cross-Adsorbed-Secondary-Antibody-Polyclonal/A-11008
Caspase-7 Antibody, https://www.cellsignal.cn/products/primary-antibodies/caspase-7-antibody/9492
Cleaved Caspase-8 (Asp374) (18C8) Rabbit mAb, https://www.cellsignal.cn/products/primary-antibodies/cleaved-caspase-8-asp374-18c8-rabbit-mab/9496
Caspase-8 (1C12) Mouse mAb, https://www.cellsignal.cn/products/primary-antibodies/caspase-8-1c12-mouse-mab/9746
Caspase-3 Antibody, https://www.cellsignal.cn/products/primary-antibodies/caspase-3-antibody/9662
MLKL (D2I6N) Rabbit mAb, https://www.cellsignal.cn/products/primary-antibodies/mlkl-d2i6n-rabbit-mab/14993
MLKL (D6W1K) Rabbit mAb, https://www.cellsignal.cn/products/primary-antibodies/mlkl-d6w1k-rabbit-mab/37705
Phospho-MLKL (Ser358) (D6H3V) Rabbit mAb, https://www.cellsignal.cn/products/primary-antibodies/phospho-mlkl-ser358-d6h3v-rabbit-mab/91689
RIP (D94C12) XP® Rabbit mAb, https://www.cellsignal.cn/products/primary-antibodies/rip-d94c12-xp-rabbit-mab/3493
Phospho-RIP (Ser166) (D1L3S) Rabbit mAb, https://www.cellsignal.cn/products/primary-antibodies/phospho-rip-ser166-d1l3s-rabbit-mab/65746
Phospho-RIP (Ser166) Antibody, https://www.cellsignal.cn/products/primary-antibodies/phospho-rip-ser166-antibody/31122
Phospho-IKKα/β (Ser176/180) (16A6) Rabbit mAb,  https://www.cellsignal.cn/products/primary-antibodies/phospho-ikka-b-ser176-180-16a6-rabbit-mab/2697
IKKα Antibody, https://www.cellsignal.com/products/primary-antibodies/ikka-antibody/2682
IKKβ (D30C6) Rabbit mAb, https://www.cellsignal.cn/products/primary-antibodies/ikkb-d30c6-rabbit-mab/8943
Phospho-NF-κB p65 (Ser536) (93H1) Rabbit mAb, https://www.cellsignal.cn/products/primary-antibodies/phospho-nf-kb-p65-ser536-93h1-rabbit-mab/3033
NF-κB p65 (D14E12) XP® Rabbit mAb, https://www.cellsignal.cn/products/primary-antibodies/nf-kb-p65-d14e12-xp-rabbit-mab/8242
TRADD (7G8) Rabbit mAb, https://www.cellsignal.cn/products/primary-antibodies/tradd-7g8-rabbit-mab/3684
c-IAP1 (D5G9) Rabbit mAb, https://www.cellsignal.cn/products/primary-antibodies/c-iap1-d5g9-rabbit-mab/7065
c-IAP2 (58C7) Rabbit mAb, https://www.cellsignal.cn/products/primary-antibodies/c-iap2-58c7-rabbit-mab/3130
TRAF2 (C192) Antibody, https://www.cellsignal.cn/products/primary-antibodies/traf2-c192-antibody/4724
Rab5 (C8B1) Rabbit mAb, https://www.cellsignal.cn/products/primary-antibodies/rab5-c8b1-rabbit-mab/3547
Cofilin (D3F9) XP® Rabbit mAb, https://www.cellsignal.cn/products/primary-antibodies/cofilin-d3f9-xp-rabbit-mab/5175
Phospho-Cofilin (Ser3) (77G2) Rabbit mAb, https://www.cellsignal.cn/products/primary-antibodies/phospho-cofilin-ser3-77g2-rabbit-mab/3313
Na+/K+-ATPase antibody (H-3), https://www.scbt.com/p/na-k-atpase-alpha-antibody-h-3?requestFrom=search

Anti-β-Actin mAb, https://www.mbl-chinawide.cn/uploads/pdf/M177-3-ver4.pdf
EFHD2 antibody, https://www.genetex.cn/Product/Detail/EFHD2-antibody/GTX108080
TNFR1 Polyclonal antibody, https://www.ptgcn.com/products/TNFR1-Antibody-21574-1-AP.htm
FLIP (D5J1E) Rabbit mAb, https://www.cellsignal.cn/products/primary-antibodies/flip-d5j1e-rabbit-mab/56343
Bcl-xL (54H6) Rabbit mAb, https://www.cellsignal.cn/products/primary-antibodies/bcl-xl-54h6-rabbit-mab/2764
Bcl-2 (D17C4) Rabbit mAb, https://www.cellsignal.cn/products/primary-antibodies/bcl-2-d17c4-rabbit-mab/3498
Monoclonal ANTI-FLAG® M2 antibody produced in mouse, https://www.sigmaaldrich.com/US/en/search/f1804?focus=products&page=1&perpage=30&sort=relevance&term=f1804&type=product

## Eukaryotic cell lines

Policy information about cell lines and Sex and Gender in Research

Cell line source(s): HCT-116 and HT-29 cell lines used in this study were obtained from Cell Resource Center, Peking Union Medical College (PCRC).

Authentication: All cell lines used in our study were authenticated by PCRC. STR profiling was used for authentication by PCRC.

Mycoplasma contamination: All cell lines were confirmed without contamination of mycoplasma.

Commonly misidentified lines (See ICLAC register): No misidentified cell lines were used.

## Palaeontology and Archaeology

Specimen provenance: *Provide provenance information for specimens and describe permits that were obtained for the work (including the name of the issuing authority, the date of issue, and any identifying information). Permits should encompass collection and, where applicable, export.*

Specimen deposition: *Indicate where the specimens have been deposited to permit free access by other researchers.*

Dating methods: *If new dates are provided, describe how they were obtained (e.g. collection, storage, sample pretreatment and measurement), where they were obtained (i.e. lab name), the calibration program and the protocol for quality assurance OR state that no new dates are provided.*

☐ Tick this box to confirm that the raw and calibrated dates are available in the paper or in Supplementary Information.

Ethics oversight: *Identify the organization(s) that approved or provided guidance on the study protocol, OR state that no ethical approval or guidance was required and explain why not.*

Note that full information on the approval of the study protocol must also be provided in the manuscript.

## Animals and other research organisms

Policy information about studies involving animals; ARRIVE guidelines recommended for reporting animal research, and Sex and Gender in Research

Laboratory animals: C57BL/6 mice obtained from Beijing Vital River Laboratory Animal Technology Co., Ltd. (Beijing, China), and the transgenic mouse lines (Efhd2f/f, Efhd2f/f Vil1cre/+, Efhd2f/f Lyzcre/+, Efhd2-/- (C57BL/6)) were bred in a specific pathogen-free environment, housed with no more than five animals per cage, and kept under controlled lighting conditions (12-hour light/12-hour dark cycle). The temperature was maintained at 24±2 °C, and humidity ranged from 40% to 70%. Both male and female mice were of 8-10 weeks during the expriments. Mice were fed a standard laboratory chow (Xietong Bio Inc., XT101FZ-002).

Wild animals: No wild animals were used.

Reporting on sex: Both male and female mice were used in our study.

Field-collected samples: No field-collected samples were used.

Ethics oversight: All animal experiments conducted in this study were carried out following the guidelines established by the Institutional Animal Care and Use Committee (IACUC) of the Institute of Laboratory Animal Science of Chinese Academy of Medical Sciences. The experimental design and procedures were reviewed and approved by the animal ethics review board (ACUC-A01-2016-006).

Note that full information on the approval of the study protocol must also be provided in the manuscript.

# Clinical data

Policy information about [clinical studies]
All manuscripts should comply with the ICMJE [guidelines for publication of clinical research] and a completed [CONSORT checklist] must be included with all submissions.

| | |
|---|---|
| Clinical trial registration | *Provide the trial registration number from ClinicalTrials.gov or an equivalent agency.* |
| Study protocol | *Note where the full trial protocol can be accessed OR if not available, explain why.* |
| Data collection | *Describe the settings and locales of data collection, noting the time periods of recruitment and data collection.* |
| Outcomes | *Describe how you pre-defined primary and secondary outcome measures and how you assessed these measures.* |

# Dual use research of concern

Policy information about [dual use research of concern]

## Hazards

Could the accidental, deliberate or reckless misuse of agents or technologies generated in the work, or the application of information presented in the manuscript, pose a threat to:

| No | Yes | |
|---|---|---|
| ☐ | ☐ | Public health |
| ☐ | ☐ | National security |
| ☐ | ☐ | Crops and/or livestock |
| ☐ | ☐ | Ecosystems |
| ☐ | ☐ | Any other significant area |

## Experiments of concern

Does the work involve any of these experiments of concern:

| No | Yes | |
|---|---|---|
| ☐ | ☐ | Demonstrate how to render a vaccine ineffective |
| ☐ | ☐ | Confer resistance to therapeutically useful antibiotics or antiviral agents |
| ☐ | ☐ | Enhance the virulence of a pathogen or render a nonpathogen virulent |
| ☐ | ☐ | Increase transmissibility of a pathogen |
| ☐ | ☐ | Alter the host range of a pathogen |
| ☐ | ☐ | Enable evasion of diagnostic/detection modalities |
| ☐ | ☐ | Enable the weaponization of a biological agent or toxin |
| ☐ | ☐ | Any other potentially harmful combination of experiments and agents |

# Plants

| | |
|---|---|
| Seed stocks | *Report on the source of all seed stocks or other plant material used. If applicable, state the seed stock centre and catalogue number. If plant specimens were collected from the field, describe the collection location, date and sampling procedures.* |
| Novel plant genotypes | *Describe the methods by which all novel plant genotypes were produced. This includes those generated by transgenic approaches, gene editing, chemical/radiation-based mutagenesis and hybridization. For transgenic lines, describe the transformation method, the number of independent lines analyzed and the generation upon which experiments were performed. For gene-edited lines, describe the editor used, the endogenous sequence targeted for editing, the targeting guide RNA sequence (if applicable) and how the editor was applied.* |
| Authentication | *Describe any authentication procedures for each seed stock used or novel genotype generated. Describe any experiments used to assess the effect of a mutation and, where applicable, how potential secondary effects (e.g. second site T-DNA insertions, mosiacism, off-target gene editing) were examined.* |

# ChIP-seq

## Data deposition

- [ ] Confirm that both raw and final processed data have been deposited in a public database such as GEO.
- [ ] Confirm that you have deposited or provided access to graph files (e.g. BED files) for the called peaks.

| | |
|---|---|
| Data access links<br>*May remain private before publication.* | *For "Initial submission" or "Revised version" documents, provide reviewer access links. For your "Final submission" document, provide a link to the deposited data.* |
| Files in database submission | *Provide a list of all files available in the database submission.* |
| Genome browser session<br>(e.g. UCSC) | *Provide a link to an anonymized genome browser session for "Initial submission" and "Revised version" documents only, to enable peer review. Write "no longer applicable" for "Final submission" documents.* |

## Methodology

| | |
|---|---|
| Replicates | *Describe the experimental replicates, specifying number, type and replicate agreement.* |
| Sequencing depth | *Describe the sequencing depth for each experiment, providing the total number of reads, uniquely mapped reads, length of reads and whether they were paired- or single-end.* |
| Antibodies | *Describe the antibodies used for the ChIP-seq experiments; as applicable, provide supplier name, catalog number, clone name, and lot number.* |
| Peak calling parameters | *Specify the command line program and parameters used for read mapping and peak calling, including the ChIP, control and index files used.* |
| Data quality | *Describe the methods used to ensure data quality in full detail, including how many peaks are at FDR 5% and above 5-fold enrichment.* |
| Software | *Describe the software used to collect and analyze the ChIP-seq data. For custom code that has been deposited into a community repository, provide accession details.* |

# Flow Cytometry

## Plots

Confirm that:

- [x] The axis labels state the marker and fluorochrome used (e.g. CD4-FITC).
- [x] The axis scales are clearly visible. Include numbers along axes only for bottom left plot of group (a 'group' is an analysis of identical markers).
- [x] All plots are contour plots with outliers or pseudocolor plots.
- [x] A numerical value for number of cells or percentage (with statistics) is provided.

## Methodology

| | |
|---|---|
| Sample preparation | Induction of apoptosis was performed using a combination of 20 ng/ml recombinant mouse or human TNF (PeproTech), 10 μg/ml CHX (Selleckchem), and 5 μM LCL-161 (MedChemExpress). To induce necroptosis, the same concentrations were used, with the addition of 20 μM zVAD-fmk (Selleckchem). At the endpoint of the experiment, cells were stained with the FITC Annexin V Apoptosis Detection Kit (BD Biosciences) following the manufacturer's instructions and analyzed using a BD LSRFortessa flow cytometer (BD Biosciences). |
| Instrument | BD LSRFortessa flow cytometer (BD Biosciences) were used for data collection in our study. |
| Software | Data were analyzed by Flowjo v10. |
| Cell population abundance | *Describe the abundance of the relevant cell populations within post-sort fractions, providing details on the purity of the samples and how it was determined.* |
| Gating strategy | Cells were first gated on FSC-A/SSC-A for excluding debris, then single cells were gated according to the boundaries between negatively and positively labeled cell populations. Unlabeled cells and single-labeled cells with each antibody were used to set boundaries. |

- [x] Tick this box to confirm that a figure exemplifying the gating strategy is provided in the Supplementary Information.

# Magnetic resonance imaging

## Experimental design

| | |
|---|---|
| Design type | *Indicate task or resting state; event-related or block design.* |
| Design specifications | *Specify the number of blocks, trials or experimental units per session and/or subject, and specify the length of each trial or block (if trials are blocked) and interval between trials.* |
| Behavioral performance measures | *State number and/or type of variables recorded (e.g. correct button press, response time) and what statistics were used to establish that the subjects were performing the task as expected (e.g. mean, range, and/or standard deviation across subjects).* |

## Acquisition

| | |
|---|---|
| Imaging type(s) | *Specify: functional, structural, diffusion, perfusion.* |
| Field strength | *Specify in Tesla* |
| Sequence & imaging parameters | *Specify the pulse sequence type (gradient echo, spin echo, etc.), imaging type (EPI, spiral, etc.), field of view, matrix size, slice thickness, orientation and TE/TR/flip angle.* |
| Area of acquisition | *State whether a whole brain scan was used OR define the area of acquisition, describing how the region was determined.* |

Diffusion MRI ☐ Used ☐ Not used

## Preprocessing

| | |
|---|---|
| Preprocessing software | *Provide detail on software version and revision number and on specific parameters (model/functions, brain extraction, segmentation, smoothing kernel size, etc.).* |
| Normalization | *If data were normalized/standardized, describe the approach(es): specify linear or non-linear and define image types used for transformation OR indicate that data were not normalized and explain rationale for lack of normalization.* |
| Normalization template | *Describe the template used for normalization/transformation, specifying subject space or group standardized space (e.g. original Talairach, MNI305, ICBM152) OR indicate that the data were not normalized.* |
| Noise and artifact removal | *Describe your procedure(s) for artifact and structured noise removal, specifying motion parameters, tissue signals and physiological signals (heart rate, respiration).* |
| Volume censoring | *Define your software and/or method and criteria for volume censoring, and state the extent of such censoring.* |

## Statistical modeling & inference

| | |
|---|---|
| Model type and settings | *Specify type (mass univariate, multivariate, RSA, predictive, etc.) and describe essential details of the model at the first and second levels (e.g. fixed, random or mixed effects; drift or auto-correlation).* |
| Effect(s) tested | *Define precise effect in terms of the task or stimulus conditions instead of psychological concepts and indicate whether ANOVA or factorial designs were used.* |

Specify type of analysis: ☐ Whole brain ☐ ROI-based ☐ Both

| | |
|---|---|
| Statistic type for inference<br>(See Eklund et al. 2016) | *Specify voxel-wise or cluster-wise and report all relevant parameters for cluster-wise methods.* |
| Correction | *Describe the type of correction and how it is obtained for multiple comparisons (e.g. FWE, FDR, permutation or Monte Carlo).* |

## Models & analysis

| n/a | Involved in the study |
|---|---|
| ☐ | ☐ Functional and/or effective connectivity |
| ☐ | ☐ Graph analysis |
| ☐ | ☐ Multivariate modeling or predictive analysis |

| | |
|---|---|
| Functional and/or effective connectivity | *Report the measures of dependence used and the model details (e.g. Pearson correlation, partial correlation, mutual information).* |
| Graph analysis | *Report the dependent variable and connectivity measure, specifying weighted graph or binarized graph, subject- or group-level, and the global and/or node summaries used (e.g. clustering coefficient, efficiency, etc.).* |
| Multivariate modeling and predictive analysis | *Specify independent variables, features extraction and dimension reduction, model, training and evaluation metrics.* |
